# The Intersection between Pharmacogenomics and Health Equity: A Case Example

**DOI:** 10.3390/pharmacy11060186

**Published:** 2023-12-05

**Authors:** Courtney Paetznick, Olihe Okoro

**Affiliations:** 1 Children’s Minnesota, Minneapolis, MN 55404, USA; courtney.paetznick@childrensmn.org; 2 Department of Pharmacy Practice and Pharmaceutical Sciences, College of Pharmacy, University of Minnesota, Duluth, MN 55812, USA

**Keywords:** pharmacogenomics, health equity, health disparities

## Abstract

Pharmacogenomics (PGx) and the study of precision medicine has substantial power to either uplift health equity efforts or further widen the gap of our already existing health disparities. In either occurrence, the medication experience plays an integral role within this intersection on an individual and population level. Examples of this intertwined web are highlighted through a case discussion. With these perspectives in mind, several recommendations for the research and clinical communities are highlighted to promote equitable healthcare with PGx integrated.

## 1. Introduction

**Figure 1:**
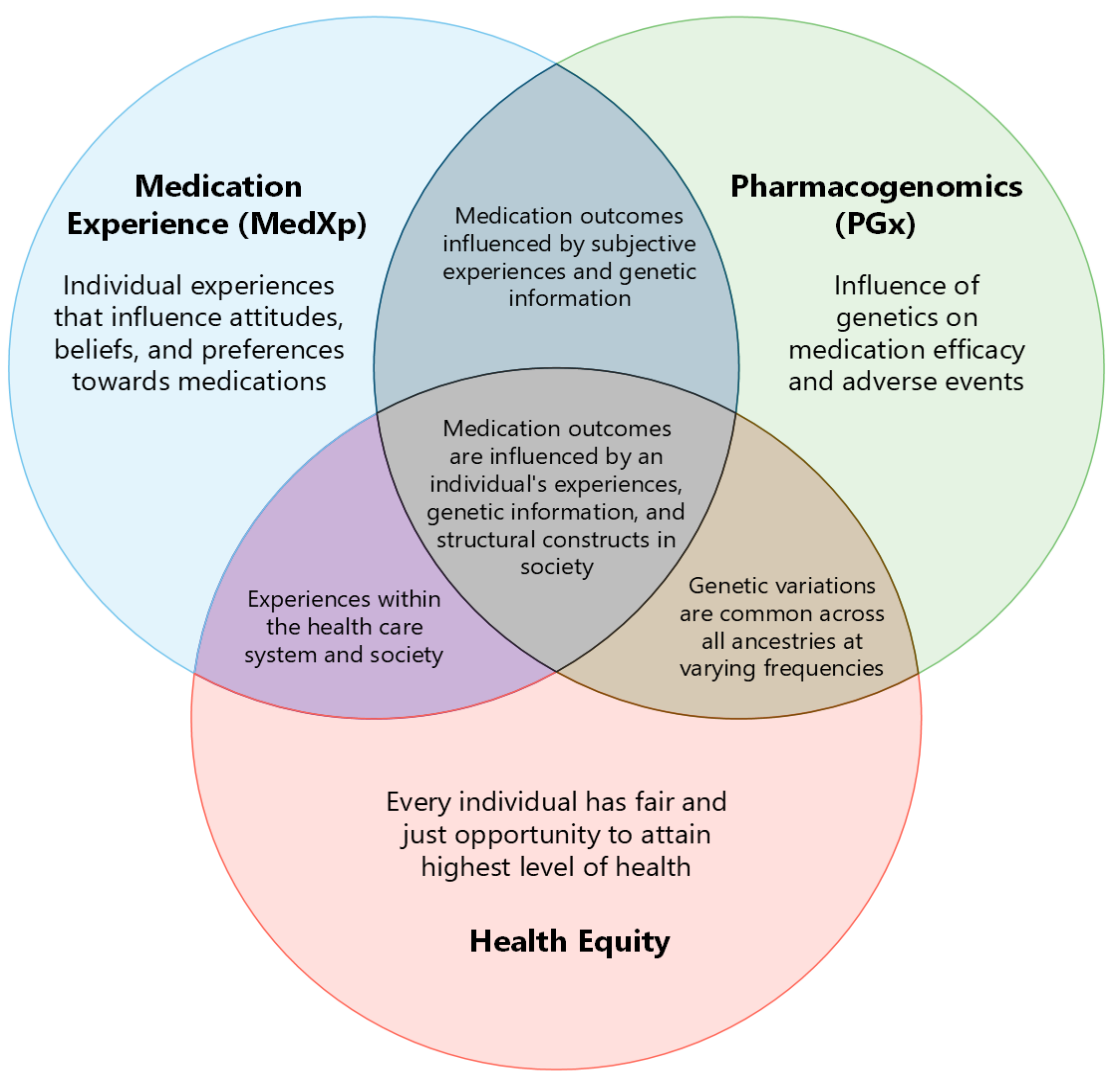
The intersection between MedXp, PGx, and health equity.

Medication experience (MedXp) is an individual’s subjective experience related to taking medication in their daily life [1]. The MedXp starts when one encounters the medication, even prior to taking it. It is inclusive of what the encounter means to them and the effects thereafter, both positive and negative. Pharmacogenomics (PGx) is a field of research that investigates how a person’s genes may affect medication response [2]. The objective of such inquiry is to optimize medication use for individuals; hence, it is integral in precision medicine. The person’s response to a medication becomes a part of their MedXp, which varies from individual to individual. Differences in MedXps and application of PGx information can contribute to inequity in health outcomes across populations. The goal of population health is to improve overall outcomes and eliminate gaps in determinants and outcomes between groups, thus achieving health equity [3]. Figure 1 highlights the interconnectedness between MedXp, PGx, and health equity. The appropriate application of PGx into patient care has the potential to enrich MedXp and influence health outcomes, allowing us to strive towards improved health equity across all biogeographical groups.

In order to better illustrate the intricacy between MedXp, PGx, and health equity, a patient story will be used as a case discussion to highlight examples of the importance of each component intersecting with one another. The purpose of this review is to focus on the intersection of PGx and health equity from the viewpoint of the MedXp. The use of an alternative name instead of initials in this case report serves as an intentional reminder of the human elements and to honor this individual’s experience and story.

## 2. Case Example

Sam is a young adult who is beginning to adjust to the idea of transferring his care from a pediatric health care system to an adult facility. Sam has sickle cell disease (HbSS) and surgical asplenia with a history of cholecystectomy, anxiety, and history of needing oxygen at home when sleeping. He has presented to the emergency department 13 times in the past year, and admitted two of those times, most commonly for chest pain. He is proud that he has been taking his hydroxyurea more consistently since his visit 2 months ago. Sam tried voxelotor in the past but was unable to tolerate it due to diarrhea. He has not had any episodes of chest pain since his last visit, with the exception of one episode associated with feeling anxious and sweating profusely. Today at his comprehensive sickle cell appointment, he also expressed interest in a maintenance anti-anxiety medication now that he feels confident in taking medications daily. Sam is currently on hydroxyzine 25 mg every 6 h as needed for anxiety, which he primarily takes at night. He feels that this is beneficial, but the benefits are not long lasting. He was previously prescribed citalopram titrated 20 mg daily by his primary care provider. He felt that citalopram at this dosage was not beneficial, and after taking it consistently for 3 months, self-discontinued it.

Sam received PGx testing as part of a research protocol one year prior. The Clinical Pharmacogenetics Implementation Consortium (CPIC) publishes peer-reviewed, evidence-based, clinical practice guidelines based on gene–drug interactions. These guidelines help with clinical decision making and standardization across institutions. For Sam, he was found to have five priority gene results that had CPIC guidelines at the time of testing (Table 1).

Self-identified race: Black/African American

 Home Medication List
 Scheduled:
  - hydroxyurea: 2000 mg by mouth daily.
  - budesonide/formoterol 160/4.5 inhalation aerosol with adapter: inhale two puffs by mouth twice daily.

 As needed:
  - acetaminophen: 650 mg by mouth every 6 h as needed for pain;
  - albuterol MDI 90 mcg/inh (CFC free) MDI: inhale two puffs by mouth every 6 h as needed for wheezing.
  - hydroxyzine hydrochloride: 25 mg by mouth every 6 h as needed for anxiety.
  - ibuprofen: 600 mg by mouth every 6 h as needed for pain;
  - polyethylene glycol 3350 oral powder: dissolve 17 g in 240 mL (8 ounces) of water or juice and drink the entire amount daily as needed for constipation.
  - oxycodone: 10 mg by mouth every 6 h as needed for severe pain. May take 5 mg instead of 10 mg if having moderate pain.

During the appointment, Sam received education from the pharmacist about the benefits and limitations of applying PGx testing into his care. The pharmacist also reviewed medications recommendations with the pediatric hematologist based on the discussion with Sam and PGx results (Table 1).With Sam transitioning to an adult hematologist soon, the pediatric hematologist recommended that he return to his primary care provider for the prescribing of anti-anxiety medication. He was strongly encouraged to bring his PGx results with him to his primary care provider and his adult hematologist when he transitioned care. The pharmacist also discussed the recommendations with the sickle cell patient health advocate who was going to be assisting Sam with care coordination and transition of care.

## 3. Discussion

### 3.1. Health Equity and MedXp

Disparities in health and healthcare persist in the United States. Groups adversely affected are characteristically racially minoritized populations whose health outcomes are further worsened by social determinants [4]. Social determinants of health (SDH) are “the non-medical factors that influence health outcomes. They are the conditions in which people are born, grow, work, live, and age, and the wider set of forces and systems shaping the conditions of daily life. These forces and systems include economic policies and systems, development agendas, social norms, social policies and political systems” [5]. Inequities in the influence and experience of these systems for different groups (referred to as systemic or structural inequities) foster socioeconomic disadvantage for minoritized populations, with a negative impact on health and well-being [6,7]. Furthermore, these SDH inevitably impact access to health care due to factors such as lack of healthcare insurance, transportation, and the cost of treatment [4]. In addition to addressing these SDH, there is a need to enhance access to care and optimize treatment for these affected groups.

While acknowledging the significant role of SDH, another systemic factor contributory to racial health disparities is race-based medicine resulting from the use of race as a biological variable versus a social construct in clinical research [8,9]. Racial categories are societal constructions based on the perceptions and interpretations of physical features and attributes, which have no direct association with biological traits [10,11]. Evidence demonstrates wide genetic variations within groups considered as racially homogenous [9,12,13]. Moreover, racial categories used in clinical studies are not consistent, and are typically assigned based on participant’s self-identification, which is subjective. In addition, the conventional racial categories used do not account for extensive racial admixture, as the world progressively becomes a global village.

Findings from clinical trials using race as biological variable have been the basis for the development of treatment guidelines and protocols, continue to undergird medical education, and consequently inform treatment decisions in patient care in areas such as cardiovascular and renal diseases [8,14]. Race-based medicine has also been shown to perpetuate provider implicit bias towards patients from minoritized groups [15].

Given this precedence, PGx as an emerging area of science must proceed with caution to avoid further contributing to racial health disparities. Sam, our patient, is from a minoritized racial group. His PGx testing results demonstrate potential benefit in optimizing his treatment for better outcomes. However, there could be further benefit if there were more PGx data representative of his ancestral origins.

### 3.2. PGx and Health Equity

PGx leverages the variation in human genome at both the individual and population level to enhance the accuracy and precision of treatment [16]. Furthermore, populations are categorized on the basis of shared ancestral origin versus self-reported “race” and “ethnicity”. Most clinical research, including the National Institute of Health (NIH), rely on race and ethnicity to describe patient demographic information. Electronic health records are also limited to documented and self-reported race and ethnicity, which can often be discordant from ancestry, causing discrepancies among genetic research studies [17]. Therefore, PGx research has advocated using biogeographical groups composed of seven geographical boundaries based on ancestral pre-diaspora and pre-colonization, and two admixed groups [18]. While biogeographical groups in PGx research have brought an awareness of equitable language and the classification of genetic variation frequencies, many PGx studies rely on self-reported methodology, which can be erroneous and flawed [19]. Similarly, the large demographic geography of the biogeographical groups can undermine subpopulation groups and regional differences that may differ vastly from the defined biographical group [20]. This presents an opportunity to bridge the racial gap in health outcomes by using biologic markers in genetics to ensure the delivery of individualized care. A standard practice of identifying DNA-based genetic ancestry is a critical next challenge in PGx research to promote equity.

Much like any new clinical implementation or change in standard of practice, the research in PGx has served as the foundation to incorporating PGx into clinical practice. While not unique to PGx research, the lack of equitable non-European representation in PGx research has led to a call to action among the PGx community [21]. The clinical evidence base for equitable genomic medicine is strengthened by increasing the diversity of populations included in genetic analysis [22,23,24]. There are disparities in participation of diverse groups in genomic studies, hence the predominance of persons of European ancestry in these studies [25,26,27,28]. There are similar trends of lack of ancestral diversity in PGx studies [29]. The lack of diversity in the study populations and thus dearth of genomic information across under-represented groups, poses a limitation to the clinical application of PGx information in therapies offered to these groups, thereby further exacerbating health disparities. Consequently, the evidence-base for precision medicine may unintentionally have greater benefit in individualizing treatment for persons who have shared common ancestry with groups of European descent [26,30].

Given the continuing challenges with lack of diversity in genomic studies, PGx present an opportunity to leverage ancestral diversity, especially in minoritized groups to improve a patients’ clinical outcomes. If an accurate and appropriate PGx test is utilized, PGx has the opportunity to minimize the risk of adverse effects, optimize doses of medications, and reduce trial-and-error approaches to treatment, which is the goal of precision medicine. Since many medications are developed and tested in clinical trials including mainly populations of European ancestry, the standard dosing approach may not be appropriate in certain patients with genetic variants that predispose the individual to increased or decreased medication exposure. With genetic variation frequencies differing in non-European ancestries, utilizing PGx may be an approach to improve medication outcomes in underserved patients.

Therefore, the push is to increase participation of under-represented groups in these studies. The key to achieving this goal is an understanding of the factors driving the lack of diversity in the populations of interest.

#### 3.2.1. Advancing the Technology

Targeted genotyping remains the leading technology in clinical PGx research due to cheaper costs and faster turnaround times than sequencing [31]. Targeted genotyping uses a set list of predefined genetic variants, which can result in misinformed results in a highly diverse patient population when the single nucleotide polymorphisms (SNPs) are genotyped based on a homogenous population group.

To demonstrate the concept, if an individual does not have any genetic variants from the targeted genotyping list, the individual gets assigned a “normal metabolizer” by default. When the list of predefined genetic variants are predominantly developed based on patients with European ancestry due to greater prevalence in research but then applied to patients of non-European ancestry, it can have significant health consequences due to potential misclassifications of their phenotype or metabolizer status. In the PGx and warfarin algorithm studies, the patients of African ancestry are an unfortunate example of inappropriate genotyping and misclassifying phenotype that resulted in higher INRs and risk of bleeding compared to European ancestry patients when using PGx-guided dosing algorithm [32,33,34].

There are challenges that can contribute to a lack of representation in designing and validating genotyping assays, including the lack of diverse reference panels, that are well described elsewhere [35]. The American College of Medical Genetics (ACMG) has curated and reviewed the evidence in order to provide recommendations for laboratories to help establish best practices [36]. In addition, the Association of Molecular Pathology (AMP) has developed a PGx working group that publishes consensus statements on the variants that should be evaluated on PGx genotyping assay panels or drug-based recommendations (e.g., warfarin) to help promote standardization. Tier 1 includes the minimum alleles and Tier 2 includes optional alleles that do not meet the criteria for Tier 1. The tier system is structured to provide guidance and is not intended to be a restrictive list. AMP plans to update the recommendations as new data or reference materials become available [37,38,39,40,41,42].

#### 3.2.2. Factors Affecting Low Participation in Research Studies

The low participation of persons from racially minoritized populations in PGx studies have been attributed to a variety of factors.

- Lack of trustworthiness of the research community and healthcare system. A major factor is the historical distrust, stemming from unethical medical practices that have had adverse transgenerational effects on the health of these populations [43,44]. The continuing experiences of systemic and interpersonal racism in the healthcare system further undermines the trustworthiness of the medical research community and healthcare system [45,46].
- Lack of diversity in the research team and healthcare workforce. Racial concordance in healthcare encounters has been associated with better patient–provider communication [46], higher likelihood of adherence to provider recommendations for preventive services such as screenings [47,48], and higher patient satisfaction with care [49], with some studies having reported better outcomes [50,51]. While there are mixed results per health outcomes based on racial concordance [52], one thing is clear: Persons from racial minoritized groups generally prefer providers with similar racial and/or ethnic background and are often more likely to follow through with recommendations when there is provider–patient concordance [53,54,55]. Likewise, investigators in a clinical trial play a key role in the recruitment and retention of study participants. Racial diversity in the research team increases social proximity to potential research participants from under-represented groups, facilitates access to hard-to-reach communities, and may make it easier to build rapport and trust [56]. Conversely, the lack of racial concordance can be a hindrance to participation by minoritized groups. Persons from these groups may be hesitant to enroll in trials if recruitment or referral is performed by personnel or providers of a different racial/ethnic background.
- Lack of referral by providers. Recruitment into PGx clinical studies is typically from points of care (clinical settings) and often through provider referrals [57]. Studies have reported an inadequate in provider knowledge of PGx, and a lack of understanding of its potential to optimize treatment [58,59,60]. This may be a major barrier to referrals for enrollment in studies or recommendations for genetic testing in general. There is also evidence of provider implicit bias in referring patients from minoritized racial groups to clinical trials [61,62]. Patients from minoritized racial groups are less likely to be referred as providers may assume that they are distrustful of research and therefore unwilling to participate. However, studies have shown that persons from these groups are as likely as their white counterparts to participate if they receive information regarding the study and its benefits and risks [63,64].

*Sam’s willingness to have PGx testing is perhaps an indication that persons from minoritized populations are not averse to genetic testing. Based on the results of his PGx test, his MedXp with lack of effectiveness from citalopram was likely influenced by his CYP2C19 rapid metabolizer status. If this information would have been available at the time of prescribing, his citalopram dosing could have been adjusted or an alternative medication could have been selected—potentially altering his MedXp. With adequate patient education on the benefits and limitations of PGx with a dedicated PGx program at Sam’s institution, the idea of improved treatment outcomes may have served as an incentive for him to participate in a PGx study. However, willingness does not equate access. How was Sam able to get his PGx test? Who ordered it? Who paid for it? Will all of his providers know how to utilize his PGx results to improve his MedXp?*

**Figure 2:**
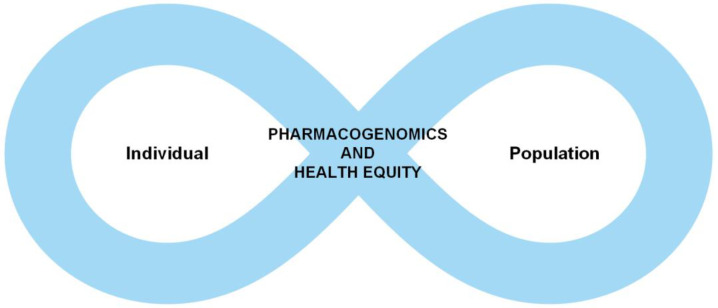
The cyclical nature of individual experiences and genetic information is shaped by health disparities and genetic information at the population level and vice versa.

There is a cyclical nature to the intersection between PGx and health equity (Figure 2). The individual experiences of minoritized persons have resulted in a lack of representation in greater numbers as a cohort. This has unfortunately misinformed PGx research and clinical application at times, that has led to significant health consequences and harm to individuals who already face adverse SDH. These traumatic experiences, then restart the cycle all over again. We must find ways to break the cycle and leverage PGx technology to uplift individuals of non-European ancestry groups rather than unintentionally increase the gap and create a further divide.

### 3.3. PGx, MedXp, and Health Equity

Currently, there is an under-utilization of recommended guidelines for PGx testing in conditions with well-established benefits, including those with potential to optimize treatment, improve health outcomes and thus help close the racial disparities [25,65]. Again, there is inequitable utilization across groups [25,65]. This is attributable to various factors including low genetic awareness and literacy, an inadequacy of knowledge among providers, and the lack of access to testing.

#### 3.3.1. Low Genetic Literacy/Lack of Awareness of Genetic Testing

There are disparities in the awareness of genetics and the relevance in medical diagnostics and treatment. In a population-based survey study, the awareness of genetic testing in cancer treatment was associated with race/ethnicity, SES, gender, beliefs and attitudes regarding cancer, and medical literacy [57,66]. Non-Hispanic white respondents had significantly higher awareness of genetics compared to other racial/ethnic groups. In another study by Tiner and Colleagues (2022), their findings demonstrated racial/ethnic- and income-based disparities in awareness of genetic testing [67]. They report that Hispanic, and non-Hispanic Asians and non-Hispanic Black respondents were less likely to be aware of or have received any type of genetic testing compared to non-Hispanic white participants [67]. In another study by Krakow et al. (2017), genetic testing awareness also differed by age, income, and race/ethnicity [68].

#### 3.3.2. Lack of Awareness and Application of PGx

PGx has the potential for significant impact on medication safety and efficacy [69] but currently has limited application in routine primary care due to the lack of awareness among healthcare providers, including physicians and pharmacists [70,71,72]. With the availability of direct-to-consumer genetic testing that does not require consultation with a healthcare provider, there has been increasing public awareness of genetic testing [68]. However, public knowledge of the applicability of genetic testing in health appears to be mostly with regard to its use in the determination of personal and hereditary disease risk, and less so for individualizing treatment and optimizing the efficacy of medications [68]. The awareness of the benefits of PGx is even less so among minoritized racial groups.

#### 3.3.3. Lack of Access to Testing

Achieving equity in adopting PGx as an integral part of routine clinical care requires that all patients have access to genetic testing. Currently, in some instances, health insurance plans will cover genetic tests if recommended by providers with justification. However, reimbursement for tests ordered are not without challenges, including determining which tests are beneficial and cost-effective. In addition, there is variation in coverage of genetic tests across health plans. Tests that may not be covered but recommended by providers to optimize treatment leaving the patient with the option of paying out of pocket or not follow the provider’s recommendation. Thus, access to PGx testing is often limited by the lack or type of insurance, as well as income level. Again, racial/ethnic groups under-represented in genomic studies are also less likely to have access to testing, as they are less likely to have insurance coverage and more likely to be of low-income status.

Another barrier to testing is the fear of insurance discrimination. There is the fear regarding privacy of data and the concern that the insurance entity could, on the basis of genetic information uncovered by the test, deny coverage or charge higher premium based on disease risk [73,74]. Such concerns are even more so for minoritized racial/ethnic groups such as African Americans, who have historical episodes of the use of medical information to perpetuate harm, intentionally [43,44]. A safeguard that has been put in place to address this barrier includes the Genetic Information Non-discrimination Act (GINA), a federal law that prohibits certain insurers from such discrimination [75]. Some states have also enacted laws that add additional protections for patients in this regard [76]. A careful consideration to minimize investigating genes related to inherited disease risk when selecting a clinical PGx test is important to mitigate these concerns.

#### 3.3.4. Ethical Considerations

Additional challenges to PGx testing are those associated with informed consent. Outside the confines of human subject research, the expectation is that PGx testing performed in a clinical setting should be treated as any other routine blood test, especially when there is empirical evidence supporting its use per specific treatment consideration. Typically, providers discuss the indications and application of a blood test with patients, and receive verbal consent to order the test. In contrast to most routine blood tests, PGx testing has the potential to return non-PGx information that may have health implications for patients and family members [65,77]. In ordering PGx tests, healthcare facilities should give careful thought to the management of the risks and benefits of incidental findings. The information to be collected and how it will be used should be explicitly spelt out and communicated to the patient in the informed consent process. Providers must also be sufficiently knowledgeable to respond to patients’ questions and concerns. Some states have legislation requiring written consent for any genetic testing, regardless of the purpose [78]. However, Spector-Bagdady and colleagues (2018), in an analysis of state legislation regarding informed consent, note that these laws tend to lack clarity and are not flexible enough to accommodate the rapid evolution of genomic technology [79]. While mandating written informed consent introduces further complexity to routinizing PGx testing in clinical care, it affords a protective layer for patients, particularly those from populations historically harmed by unethical conduct of medical research and medical practice [80].

*As discussed, several factors are implicated in Sam’s access to PGx testing and consequently its utilization in optimizing his treatment. Fortunately, Sam had access to PGx testing through grant funding that focused on PGx in individuals with sickle cell disease. Now that Sam is transitioning between health care facilities, the sickle cell patient-family advocate has a critical role in assisting with this transition. Therefore, PGx education should not only take place with Sam, but also with the health care team, including the health advocate, so they can adequately support Sam’s MedXp by advocating that his new providers become accustomed to using his PGx results. Considerations for these factors and intentionally addressing those that are barriers, not only benefits Sam and his MedXp, but also populations currently underrepresented in PGx.*

## 4. Recommendations

There are many potential ideas and recommendations that are drawn from this discussion to improve exploratory and clinical research in PGx to optimize and promote health equity efforts. A few to highlight are as follows.

Given the highly variable ancestry admixtures within racial/ethnic populations, there is a need to have data representative of the whole population spectrum. While careful consideration must be given to the inclusion of all racial/ethnic groups, it is critical that categorizations be made exclusively using genetically determined ancestry, not race/ethnicity [81].

It is important to increase non-European ancestry-specific genome-wide association studies (GWAS) studies to identify variants not seen in European ancestry [82]. To achieve adequate representation in PGx studies and thus strengthen the evidence base for the use of genomic data in optimizing therapies, more targeted efforts must be made to recruit participants from under-represented groups. An effective recruitment approach is community engagement, which helps build trust with under-represented and historically marginalized communities and thus encourages participation [83]. Per the ethos of community engagement, efforts should prioritize relationship-building and seek partnerships with these communities at all phases of research, and not only at recruitment.

With the rapid growth in the field of PGx, it is important to consider the lessons learned with previous clinical studies and ensure that race is not used as a proxy for clinical decision making, which would further perpetuate health disparities. Rather than rely on race/ethnicity as a differentiator, PGx research should adopt the standard practice of identifying DNA-based genetic ancestry as it moves towards the use of biogeographical groups established based on ancestral pre-diaspora and pre-colonization, and admixture [18]. Global standardization based on biological variables will ensure that the clinical application of PGx information is not race-based, but rather help determine where there might be gaps per benefits for various racial groups. Policies and laws must continue to evolve to provide protections against the discrimination and mishandling of genetically based information regarding ancestry and PGx results.

Emphasis should be placed on provider education and training. Integrating PGx in routine clinical care will require adequate provider training and education in interpretation and implementation [72]. Given the ethical concerns around genomic information and its use, providers also need to receive training on obtaining informed consent from patients and the management of incidental non-PGx data. Appropriate protocols must be put in place regarding the use of PGx data.

To address provider implicit bias in referrals and recommendations, requisite training should not only address these biases, but seek to build cultural competency, which generally refers to the ability of providers and healthcare entities to provide care to patients with diverse values, beliefs and behaviors, including tailoring health care delivery to effectively meet these patients’ sociocultural and linguistic needs [84].

Health policies should ensure equitable insurance coverage of PGx testing for all patients regardless of socioeconomic status. Given that minoritized racial/ethnic groups are more adversely affected by socioeconomic disparities [85], any inequity in access to PGx testing, which includes cost, will further contribute to the racial health disparities that persist in the US.

The trustworthiness of the medical community and researchers remains a barrier to the engagement of minoritized racial groups in clinical research and genetic testing in patient care. Efforts should be made to increase the diversity of the healthcare workforce and ensure adequate representation among research investigators and other personnel. This requires more broadly addressing inequities in education and associated cost that result in under-representation in health professions [86,87].

If targeted genotyping is utilized for PGx testing, laboratories should consider the best practices put forth by ACMG [36]. In addition, clinical PGx application should ensure that the PGx laboratory includes Tier 1 and preferably Tier 2 allele recommendations put forth by the AMP PGx working group if available for the gene(s) of interest [37,38,39,40,41,42]. Ideally, the sequencing of pharmacogenes before medication therapy is required will become the standard of care in order to minimize the clinical impact of slower turnaround time with sequencing and mitigate health equity concerns with genotyping [88,89].

Apply an equity lens. PGx research authors and publication reviewers should critically examine the research cohort to identify missing genetic variants in the evaluation that are known and may confound outcomes in non-European ancestry groups [90].

## 5. Conclusions

PGx is an effective tool in optimizing treatment and presents an opportunity to close the racial health disparities that persist. However, the under-representation of minoritized racial populations in PGx clinical studies and barriers to access in PGx testing in clinical care are ongoing challenges to achieving health equity in this regard. Efforts should be made to increase the enrollment of populations of non-European ancestry in clinical studies to strengthen the evidence base for the use of genomic data in optimizing therapies in these populations.

## Figures and Tables

**Table 1:** Sam’s priority PGx results from the PGx research protocol.

Gene	Diplotype	Phenotype
CYP2B6	*6/*6	Poor Metabolizer
CYP2C19	*1/*17	Rapid Metabolizer
CYP2D6	*1/*5	Intermediate Metabolizer Activity Score = 1
CYP3A5	*1/*3	Intermediate Metabolizer
UGT1A1	*1/*28 + 80, *28/*80	Intermediate Metabolizer

The * is a standard nomenclature used to describe PGx haplotype genetic results.

## Data Availability

Not applicable.

## References

[1] Shoemaker S.J., Ramalho de Oliveira D. (2008). Understanding the meaning of medications for patients: The medication experience. Pharm. World Sci..

[2] The National Institute of General Medical Sciences Pharmacogenomics. Last Updated 4 May 2022. https://nigms.nih.gov/education/fact-sheets/Pages/pharmacogenomics.aspx.

[3] Kindig D. (2017). Population Health Equity: Rate and Burden, Race and Class. JAMA.

[4] Ndugga N., Artiga S. Disparities in Health and Health Care: 5 Key Questions and Answers. KFF. Online published 21 April 2023. https://www.kff.org/racial-equity-and-health-policy/issue-brief/disparities-in-health-and-health-care-5-key-question-and-answers/view/footnotes/.

[5] World Health Organization Social Determinants of Health. https://www.who.int/health-topics/social-determinants-of-health#tab=tab_1.

[6] Churchwell K., Elkind M.S.V., Benjamin R.M., Carson A.P., Chang E.K., Lawrence W., Mills A., Odom T.M., Rodriguez C.J., Rodriguez F. (2020). Call to Action: Structural Racism as a Fundamental Driver of Health Disparities: A Presidential Advisory from the American Heart Association. Circulation.

[7] Bailey Z.D., Feldman J.M., Bassett M.T. (2021). How Structural Racism Works—Racist Policies as a Root Cause of U.S. Racial Health Inequities. N. Engl. J. Med..

[8] Cerdeña J.P., Plaisime M.V., Tsai J. (2020). From race-based to race-conscious medicine: How anti-racist uprisings call us to act. Lancet.

[9] Amutah C., Greenidge K., Mante A., Munyikwa M., Surya S.L., Higginbotham E., Jones D.S., Lavizzo-Mourey R., Roberts D., Tsai J. (2021). Misrepresenting Race—The Role of Medical Schools in Propagating Physician Bias. N. Engl. J. Med..

[10] Saperstein A., Penner A.M., Light R. (2013). Racial Formation in Perspective: Connecting Individuals, Institutions, and Power Relations. Annu. Rev. Sociol.

[11] Nelson D.L., Korf B.R. (2018). ASHG Perspectives: A new Voice for ASHG. Am. J. Hum. Genet..

[12] Witherspoon D.J., Wooding S., Rogers A.R., Marchani E.E., Watkins W.S., Batzer M.A., Jorde L.B. (2007). Genetic similarities within and between human populations. Genetics.

[13] Collins R.L., Brand H., Karczewski K.J., Zhao X., Alföldi J., Francioli L.C., Khera A.V., Lowther C., Gauthier L.D., Wang H. (2020). A structural variation reference for medical and population genetics. Nature.

[14] Parsons S. (2020). Addressing Racial Biases in Medicine: A Review of the Literature, Critique, and Recommendations. Int. J. Health Serv..

[15] Okoro O.N., Arya V., Gaither C.A., Tarfa A. (2021). Examining the Inclusion of Race and Ethnicity in Patient Cases. Am. J. Pharm. Educ..

[16] Popejoy A.B. (2019). Diversity in Precision Medicine and Pharmacogenetics: Methodological and Conceptual Considerations for Broadening Participation. Pharmgenom. Pers. Med..

[17] Samalik J.M., Goldberg C.S., Modi Z.J., Fredericks E.M., Gadepalli S.K., Eder S.J., Adler J. (2022). Discrepancies in Race and Ethnicity in the Electronic Health Record Compared to Self-report. J. Racial Ethn. Health Disparities.

[18] Huddart R., Fohner A.E., Whirl-Carrillo M., Wojcik G.L., Gignoux C.R., Popejoy A.B., Bustamante C.D., Altman R.B., Klein T.E. (2019). Standardized Biogeographic Grouping System for Annotating Populations in Pharmacogenetic Research. Clin. Pharmacol. Ther..

[19] Mersha T.B., Abebe T. (2015). Self-reported race/ethnicity in the age of genomic research: Its potential impact on understanding health disparities. Hum. Genom..

[20] Suarez-Kurtz G., Pena S.D., Struchiner C.J., Hutz M.H. (2012). Pharmacogenomic Diversity among Brazilians: Influence of Ancestry, Self-Reported Color, and Geographical Origin. Front. Pharmacol..

[21] Luczak T., Stenehjem D., Brown J. (2021). Applying an equity lens to pharmacogenetic research and translation to under-represented populations. Clin. Transl. Sci..

[22] Hindorff L.A., Bonham V.L., Brody L.C., Ginoza M.E.C., Hutter C.M., Manolio T.A., Green E.D. (2018). Prioritizing diversity in human genomics research. Nat. Rev. Genet..

[23] Wojcik G.L., Graff M., Nishimura K.K., Tao R., Haessler J., Gignoux C.R., Highland H.M., Patel Y.M., Sorokin E.P., Avery C.L. (2019). Genetic analyses of diverse populations improves discovery for complex traits. Nature.

[24] Alrajeh K.Y., Roman Y.M. (2022). The frequency of major *CYP2C19* genetic polymorphisms in women of Asian, Native Hawaiian and Pacific Islander subgroups. Per. Med..

[25] Mamun A., Nsiah N.Y., Srinivasan M., Chaturvedula A., Basha R., Cross D., Jones H.P., Nandy K., Vishwanatha J.K. (2019). Diversity in the Era of Precision Medicine—From Bench to Bedside Implementation. Ethn. Dis..

[26] Sirugo G., Williams S.M., Tishkoff S.A. (2019). The Missing Diversity in Human Genetic Studies. Cell.

[27] Gurdasani D., Barroso I., Zeggini E., Sandhu M.S. (2019). Genomics of disease risk in globally diverse populations. Nat. Rev. Genet..

[28] Graham S.E., Clarke S.L., Wu K.H., Kanoni S., Zajac G.J.M., Ramdas S., Surakka I., Ntalla I., Vedantam S., Winkler T.W. (2021). The power of genetic diversity in genome-wide association studies of lipids. Nature.

[29] Zhang H., De T., Zhong Y., Perera M.A. (2019). The Advantages and Challenges of Diversity in Pharmacogenomics: Can Minority Populations Bring Us Closer to Implementation?. Clin. Pharmacol. Ther..

[30] Popejoy A.B., Fullerton S.M. (2016). Genomics is failing on diversity. Nature.

[31] Schwarz U.I., Gulilat M., Kim R.B. (2019). The Role of Next-Generation Sequencing in Pharmacogenetics and Pharmacogenomics. Cold Spring Harb. Perspect Med..

[32] Drozda K., Wong S., Patel S.R., Bress A.P., Nutescu E.A., Kittles R.A., Cavallari L.H. (2015). Poor warfarin dose prediction with pharmacogenetic algorithms that exclude genotypes important for African Americans. Pharmacogenet. Genom..

[33] Kimmel S.E., French B., Kasner S.E., Johnson J.A., Anderson J.L., Gage B.F., Rosenberg Y.D., Eby C.S., Madigan R.A., McBane R.B. (2013). A pharmacogenetic versus a clinical algorithm for warfarin dosing. N. Engl. J. Med..

[34] Pirmohamed M., Burnside G., Eriksson N., Jorgensen A.L., Toh C.H., Nicholson T., Kesteven P., Christersson C., Wahlström B., Stafberg C. (2013). A randomized trial of genotype-guided dosing of warfarin. N. Engl. J. Med..

[35] Scionti F., Pensabene L., Di Martino M.T., Arbitrio M., Tagliaferri P. (2022). Ethical Perspectives on Pharmacogenomic Profiling. Compr. Pharmacol..

[36] Tayeh M.K., Gaedigk A., Goetz M.P., Klein T.E., Lyon E., McMillin G.A., Rentas S., Shinawi M., Pratt V.M., Scott S.A. (2022). Clinical pharmacogenomic testing and reporting: A technical standard of the American College of Medical Genetics and Genomics (ACMG). Genet. Med..

[37] Pratt V.M., Del Tredici A.L., Hachad H., Ji Y., Kalman L.V., Scott S.A., Weck K.E. (2018). Recommendations for Clinical CYP2C19 Genotyping Allele Selection: A Report of the Association for Molecular Pathology. J. Mol. Diagn..

[38] Pratt V.M., Cavallari L.H., Del Tredici A.L., Hachad H., Ji Y., Moyer A.M., Scott S.A., Whirl-Carrillo M., Weck K.E. (2019). Recommendations for Clinical CYP2C9 Genotyping Allele Selection: A Joint Recommendation of the Association for Molecular Pathology and College of American Pathologists. J. Mol. Diagn..

[39] Pratt V.M., Cavallari L.H., Del Tredici A.L., Hachad H., Ji Y., Kalman L.V., Ly R.C., Moyer A.M., Scott S.A., Whirl-Carrillo M. (2020). Recommendations for Clinical Warfarin Genotyping Allele Selection: A Report of the Association for Molecular Pathology and the College of American Pathologists. J. Mol. Diagn..

[40] Pratt V.M., Cavallari L.H., Del Tredici A.L., Gaedigk A., Hachad H., Ji Y., Kalman L.V., Ly R.C., Moyer A.M., Scott S.A. (2021). Recommendations for Clinical CYP2D6 Genotyping Allele Selection: A Joint Consensus Recommendation of the Association for Molecular Pathology, College of American Pathologists, Dutch Pharmacogenetics Working Group of the Royal Dutch Pharmacists Association, and the European Society for Pharmacogenomics and Personalized Therapy. J. Mol. Diagn..

[41] Pratt V.M., Cavallari L.H., Fulmer M.L., Gaedigk A., Hachad H., Ji Y., Kalman L.V., Ly R.C., Moyer A.M., Scott S.A. (2022). TPMT and NUDT15 Genotyping Recommendations: A Joint Consensus Recommendation of the Association for Molecular Pathology, Clinical Pharmacogenetics Implementation Consortium, College of American Pathologists, Dutch Pharmacogenetics Working Group of the Royal Dutch Pharmacists Association, European Society for Pharmacogenomics and Personalized Therapy, and Pharmacogenomics Knowledgebase. J. Mol. Diagn..

[42] Pratt V.M., Cavallari L.H., Fulmer M.L., Gaedigk A., Hachad H., Ji Y., Kalman L.V., Ly R.C., Moyer A.M., Scott S.A. (2023). CYP3A4 and CYP3A5 Genotyping Recommendations: A Joint Consensus Recommendation of the Association for Molecular Pathology, Clinical Pharmacogenetics Implementation Consortium, College of American Pathologists, Dutch Pharmacogenetics Working Group of the Royal Dutch Pharmacists Association, European Society for Pharmacogenomics and Personalized Therapy, and Pharmacogenomics Knowledgebase. J. Mol. Diagn..

[43] Smirnoff M., Wilets I., Ragin D.F., Adams R., Holohan J., Rhodes R., Winkel G., Ricci E.M., Clesca C., Richardson L.D. (2018). A paradigm for understanding trust and mistrust in medical research: The Community VOICES study. AJOB Empir. Bioeth..

[44] Scharff D.P., Mathews K.J., Jackson P., Hoffsuemmer J., Martin E., Edwards D. (2010). More than Tuskegee: Understanding mistrust about research participation. J. Health Care Poor Underserved.

[45] Bazargan M., Cobb S., Assari S. (2021). Discrimination and Medical Mistrust in a Racially and Ethnically Diverse Sample of California Adults. Ann. Fam. Med..

[46] Thompson H.S., Manning M., Mitchell J., Kim S., Harper F.W.K., Cresswell S., Johns K., Pal S., Dowe B., Tariq M. (2021). Factors Associated With Racial/Ethnic Group-Based Medical Mistrust and Perspectives on COVID-19 Vaccine Trial Participation and Vaccine Uptake in the US. JAMA Netw. Open.

[47] Jetty A., Jabbarpour Y., Pollack J., Huerto R., Woo S., Petterson S. (2022). Patient-Physician Racial Concordance Associated with Improved Healthcare Use and Lower Healthcare Expenditures in Minority Populations. J. Racial Ethn. Health Disparit..

[48] Marcella A., Garrick O., Graziani G. (2019). Does Diversity Matter for Health? Experimental Evidence from Oakland. Am. Econ. Rev..

[49] Ku L., Vichare A. (2023). The Association of Racial and Ethnic Concordance in Primary Care with Patient Satisfaction and Experience of Care. J. Gen. Intern. Med..

[50] Cooper L.A., Powe N.R. (2004). Disparities in Patient Experiences, Health Care Processes, and Outcomes: The Role of Patient-Provider Racial, Ethnic, and Language Concordance. Commonwealth Fund. https://www.commonwealthfund.org/publications/fund-reports/2004/jul/disparities-patient-experiences-health-care-processes-and.

[51] Lightfoot E., McCleary J.S., Lum T. (2004). Asset Mapping as a Research Tool for Community-Based Participatory Research in Social Work. Soc. Work Res..

[52] Meghani S.H., Brooks J.M., Gipson-Jones T., Waite R., Whitfield-Harris L., Deatrick J.A. (2009). Patient-provider race-concordance: Does it matter in improving minority patients’ health outcomes?. Ethn. Health.

[53] Street R.L., O’Malley K.J., Cooper L.A., Haidet P. (2008). Understanding concordance in patient-physician relationships: Personal and ethnic dimensions of shared identity. Ann. Fam. Med..

[54] García J.A., Paterniti D.A., Romano P.S., Kravitz R.L. (2003). Patient Preferences for Physician Characteristics in University-Based Primary Care Clinics. Ethn. Dis..

[55] Cooper L.A., Beach M.C., Johnson R.L., Inui T.S. (2006). Delving below the surface. Understanding how race and ethnicity influence relationships in health care. J. Gen. Intern. Med..

[56] Hattery A.J., Smith E., Magnuson S., Monterrosa A., Kafonek K., Shaw C., Mhonde R.D., Kanewske L.C. (2022). Diversity, Equity, and Inclusion in Research Teams: The Good, The Bad, and The Ugly. Race Justice.

[57] Rohrer Vitek C.R., Abul-Husn N.S., Connolly J.J., Hartzler A.L., Kitchner T., Peterson J.F., Rasmussen L.V., Smith M.E., Stallings S., Williams M.S. (2017). Healthcare provider education to support integration of pharmacogenomics in practice: The eMERGE Network experience. Pharmacogenomics.

[58] Just K.S., Steffens M., Swen J.J., Patrinos G.P., Guchelaar H.J., Stingl J.C. (2017). Medical education in pharmacogenomics-results from a survey on pharmacogenetic knowledge in healthcare professionals within the European pharmacogenomics clinical implementation project Ubiquitous Pharmacogenomics (U-PGx). Eur. J. Clin. Pharmacol..

[59] Haga S.B. (2017). Educating patients and providers through comprehensive pharmacogenetic test reports. Pharmacogenomics.

[60] Borden B.A., Galecki P., Wellmann R., Danahey K., Lee S.M., Patrick-Miller L., Sorrentino M.J., Nanda R., Koyner J.L., Polonsky T.S. (2019). Assessment of provider-perceived barriers to clinical use of pharmacogenomics during participation in an institutional implementation study. Pharmacogenet. Genom..

[61] Niranjan S.J., Martin M.Y., Fouad M.N., Vickers S.M., Wenzel J.A., Cook E.D., Konety B.R., Durant R.W. (2020). Bias and stereotyping among research and clinical professionals: Perspectives on minority recruitment for oncology clinical trials. Cancer.

[62] Durant R.W., Wenzel J.A., Scarinci I.C., Paterniti D.A., Fouad M.N., Hurd T.C., Martin M.Y. (2014). Perspectives on barriers and facilitators to minority recruitment for clinical trials among cancer center leaders, investigators, research staff, and referring clinicians: Enhancing minority participation in clinical trials (EMPaCT). Cancer.

[63] Patel A.P., Paranjpe M.D., Kathiresan N.P., Rivas M.A., Khera A.V. (2020). Race, socioeconomic deprivation, and hospitalization for COVID-19 in English participants of a national biobank. Int. J. Equity Health.

[64] Byrne M.M., Tannenbaum S.L., Glück S., Hurley J., Antoni M. (2014). Participation in cancer clinical trials: Why are patients not participating?. Med. Decis. Mak..

[65] Hippman C., Nislow C. (2019). Pharmacogenomic Testing: Clinical Evidence and Implementation Challenges. J. Pers. Med..

[66] Giri V.N., Shimada A., Leader A.E. (2021). Predictors of Population Awareness of Cancer Genetic Tests: Implications for Enhancing Equity in Engaging in Cancer Prevention and Precision Medicine. JCO Precis. Oncol..

[67] Tiner J.C., Mechanic L.E., Gallicchio L., Gillanders E.M., Helzlsouer K.J. (2022). Awareness and use of genetic testing: An analysis of the Health Information National Trends Survey 2020. Genet. Med..

[68] Krakow M., Ratcliff C.L., Hesse B.W., Greenberg-Worisek A.J. (2017). Assessing Genetic Literacy Awareness and Knowledge Gaps in the US Population: Results from the Health Information National Trends Survey. Public Health Genom..

[69] Carr D.F., Alfirevic A., Pirmohamed M. (2014). Pharmacogenomics: Current State-of-the-Art. Genes.

[70] Rigter T., Jansen M.E., de Groot J.M., Janssen S.W.J., Rodenburg W., Cornel M.C. (2020). Implementation of Pharmacogenetics in Primary Care: A Multi-Stakeholder Perspective. Front. Genet..

[71] Haga S.B., Burke W., Ginsburg G.S., Mills R., Agans R. (2012). Primary care physicians’ knowledge of and experience with pharmacogenetic testing. Clin. Genet..

[72] French E.L., Kader L., Young E.E., Fontes J.D. (2023). Physician Perception of the Importance of Medical Genetics and Genomics in Medical Education and Clinical Practice. Med. Educ. Online.

[73] Lee D.C., Liang H., Shi L. (2021). The convergence of racial and income disparities in health insurance coverage in the United States. Int. J. Equity Health.

[74] Fulda K.G., Lykens K. (2006). Ethical issues in predictive genetic testing: A public health perspective. J. Med. Ethics.

[75] Slaughter L.M. (2013). Getting the word out on GINA. Am. Nurse.

[76] National Conference of State Legislatures. https://www.ncsl.org/.

[77] Gershon E.S., Alliey-Rodriguez N., Grennan K. (2014). Ethical and public policy challenges for pharmacogenomics. Dialogues Clin. Neurosci..

[78] National Human Genome Research Institute Genome Statute and Legislation Database. https://www.genome.gov/about-genomics/policy-issues/Genome-Statute-Legislation-Database?page.

[79] Spector-Bagdady K., Prince A.E.R., Yu J.H., Appelbaum P.S. (2018). Analysis of state laws on informed consent for clinical genetic testing in the era of genomic sequencing. Am. J. Med. Genet. C Semin. Med. Genet..

[80] Washington H.A. (2006). Medical Apartheid: The Dark History of Medical Experimentation on Black Americans from Colonial Times to the Present.

[81] Magavern E.F., Gurdasani D., Ng F.L., Lee S.S. (2022). Health equality, race and pharmacogenomics. Br. J. Clin. Pharmacol..

[82] Suarez-Kurtz G. (2021). Population impact of pharmacogenetic tests in admixed populations across the Americas. Pharmacogenom. J..

[83] Holzer J.K., Ellis L., Merritt M.W. (2014). Why we need community engagement in medical research. J. Investig. Med..

[84] Health Research & Educational Trust, Institute for Diversity in Health Management (2011). Building a Culturally Competent Organization: The Quest for Equity in Health Care.

[85] Williams D.R., Mohammed S.A., Leavell J., Collins C. (2010). Race, socioeconomic status, and health: Complexities, ongoing challenges, and research opportunities. Ann. N. Y. Acad. Sci..

[86] Gonzalez D., Kenney G.M., McDaniel M., O’Brien C. (2022). Racial, Ethnic, and Language Concordance between Patients and Their Usual Health Care Providers.

[87] Kang Y., Ibrahim S.A. (2020). Debt-Free Medical Education-A Tool for Health Care Workforce Diversity. JAMA Health Forum.

[88] Richards S., Aziz N., Bale S., Bick D., Das S., Gastier-Foster J., Grody W.W., Hegde M., Lyon E., Spector E. (2015). Standards and guidelines for the interpretation of sequence variants: A joint consensus recommendation of the American College of Medical Genetics and Genomics and the Association for Molecular Pathology. Genet. Med..

[89] Wang L., Scherer S.E., Bielinski S.J., Muzny D.M., Jones L.A., Black J.L., Moyer A.M., Giri J., Sharp R.R., Matey E.T. (2022). Implementation of preemptive DNA sequence-based pharmacogenomics testing across a large academic medical center: The Mayo-Baylor RIGHT 10K Study. Genet. Med..

[90] Kabbani D., Akika R., Wahid A., Daly A.K., Cascorbi I., Zgheib N.K. (2023). Pharmacogenomics in practice: A review and implementation guide. Front. Pharmacol..

